# A practical framework for interpreting intraoperative electrophysiological signals in acoustic neuroma surgery

**DOI:** 10.1016/j.cnp.2026.09.006

**Published:** 2026-09-29

**Authors:** Juan Li, Qing Liu, Xianrui Yuan, Jian Yuan

**Affiliations:** Department of Neurosurgery, Xiangya Hospital, Central South University, Changsha 410008, China

**Keywords:** Acoustic neuroma, Facial nerve monitoring, Intraoperative neurophysiological monitoring, Predictive thresholds

## Abstract

**Objective:**

To develop a practical, stepwise framework for interpreting intraoperative neurophysiological signals to predict facial nerve outcomes after acoustic neuroma surgery.

**Methods:**

This methodological report synthesizes evidence from three IONM modalities: facial motor evoked potentials (FMEPs), direct electrical stimulation (DES), and free-running electromyography (EMG). Key predictive parameters, threshold values, sensitivity, specificity, and technical limitations were extracted from published meta-analyses, systematic reviews, and prospective cohort studies. These parameters were then organized chronologically into a stepwise interpretation framework according to their typical intraoperative use.

**Results:**

An end-of-surgery FMEP amplitude reduction >50% predicts poor long-term (≥1 year) facial function with 93% specificity. A DES threshold ≤0.05 mA at the brainstem stump predicts good long-term function (73% sensitivity, 74% specificity). A cumulative A-train duration >10 s on free-running EMG signals high risk for severe immediate palsy (66.7% sensitivity, 88.0% specificity). The 2026 CNS guidelines confirm that electrophysiological parameters predict long-term outcomes better than clinical information alone.

**Conclusions:**

This report provides a clinically usable, stepwise framework that integrates FMEP, DES, and free-running EMG thresholds for intraoperative prediction of facial nerve function.

**Significance:**

Despite routine use of intraoperative neurophysiological monitoring, no standardized framework has existed to translate real-time signals into postoperative facial nerve predictions. This framework fills that gap by offering surgeons and monitoring teams a clear, evidence-based, and chronologically ordered decision aid, potentially improving early recognition of poor outcomes and facilitating timely referral for facial reanimation.

## Highlights

• Facial motor evoked potential (FMEP) drop >50% predicts poor outcome.
• Direct stimulation (DES) threshold ≤0.05 mA at brainstem predicts good function.
• Framework integrates FMEP, DES, and free-running EMG intraoperatively.

## 1 Introduction

Acoustic neuromas (vestibular schwannomas) are the most common benign tumors of the cerebellopontine angle. Over the past decade, the treatment goal has shifted from complete resection to tumor control with functional preservation (Carlson and Link, 2021). Facial nerve palsy remains a major complication, causing disfigurement, eye closure problems, anxiety, and reduced quality of life (Saadi et al., 2019).

Intraoperative neurophysiological monitoring improves facial nerve outcomes (Delgado et al., 1979). The 2026 Congress of Neurological Surgeons (CNS) guidelines state that intraoperative facial nerve monitoring provides better long-term outcomes than anatomical dissection alone and recommend it as routine in all acoustic neuroma cases (Patel et al., 2026). The guidelines also note that electrophysiological parameters predict long-term facial nerve outcomes better than clinical information alone.

The question has shifted from whether monitoring helps to whether intraoperative signals can predict recovery. Accurate prediction helps identify patients likely to develop severe palsy, allowing timely planning of secondary facial nerve reconstruction (Freeman et al., 2025).

Existing guidelines support IONM use (Kartush et al., 2021; Patel et al., 2026), but none provide a stepwise framework for intraoperative prediction. The literature remains fragmented: studies use different thresholds, outcome measures, and time points (Zachem et al., 2025). Although a systematic review has summarized the evidence for individual modalities (Acioly et al., 2013), no practical framework yet combines them for real-time intraoperative use. This report develops a stepwise interpretation framework that synthesizes three monitoring modalities as a clinical reference.

## 2 Methods

This methodological report followed a structured evidence synthesis approach and is not a systematic review.

### 2.1 Literature search and selection

We searched PubMed, Embase, Web of Science, and CNKI from inception through March 2026 using combinations of the following terms: “acoustic neuroma” OR “vestibular schwannoma” AND “facial nerve” AND (“IONM” OR “motor evoked potential” OR “direct electrical stimulation” OR “free-running EMG” OR “A-train”). No language restrictions were applied.

Meta-analyses, systematic reviews, and prospective cohort studies that reported clearly defined threshold values for predicting facial nerve outcomes were prioritized. Case reports, technical notes without outcome data, and studies without explicit threshold definitions were excluded. From eligible studies we extracted: (1) the monitoring parameter, (2) the threshold defining good versus poor outcome, (3) sensitivity and specificity when available, and (4) technical limitations.

### 2.2 Parameter selection

Three IONM modalities were chosen because they complement each other: FMEPs (global pathway integrity), DES (focal anatomical and functional integrity), and free-running EMG (real-time irritative monitoring). For each modality, the most reliable predictive parameter and its corresponding threshold were identified from the highest available evidence level (meta-analysis > prospective cohort > retrospective study).

### 2.3 Framework construction

The three modalities were arranged chronologically according to their typical intraoperative timing. A stepwise algorithm was then constructed: (1) FMEP monitoring during tumor dissection (early warning), (2) DES assessment after tumor removal (focal verification), and (3) free-running EMG review throughout surgery (real-time history). The framework combines findings from all three modalities into an overall interpretation, as summarized in Table 1 and detailed in the Results section. Fig. 1 provides a visual decision aid for the combined interpretation.

**Table 1:** Predictive Thresholds and Clinical Application of Three Monitoring Modalities.

Modality	Key parameter	Threshold for good outcome	Threshold for poor outcome	Primary advantage	Primary limitation
**FMEPs**	End-surgery amplitude ratio (final/baseline)	Decrease <50%	Decrease >50% (93% specificity) (Pan et al., 2024)	Early warning, global pathway assessment	Anesthetic effects; insensitive to distal focal injury
**DES**	Minimum stimulation threshold (brainstem stump)	≤0.05 mA (73% sensitivity, 74% specificity) (Quimby et al., 2021)	>0.10 mA	Focal integrity, anatomical and functional information	Nerve edema may cause false positive; wait 10–15 min before final assessment
**Free-running EMG**	Cumulative A-train duration (“train time”)	<0.5 s (Prell et al., 2007)	>10 s (66.7% sensitivity, 88.0% specificity) (Prell et al., 2007)	Real-time warning, continuous monitoring	Subjective pattern recognition; silent injury possible; non-linear relationship between train time and palsy severity (Prell et al., 2014)

Abbreviations: mA, milliampere; s, second.

Notes: Sensitivity and specificity values for FMEPs from Pan et al., 2024; for DES from Quimby et al., 2021. A-train sensitivity and specificity for prediction of immediate postoperative palsy from Prell et al., 2007. The non-linear relationship between train time and palsy severity was described by Prell et al., 2014.

**Fig. 1:**
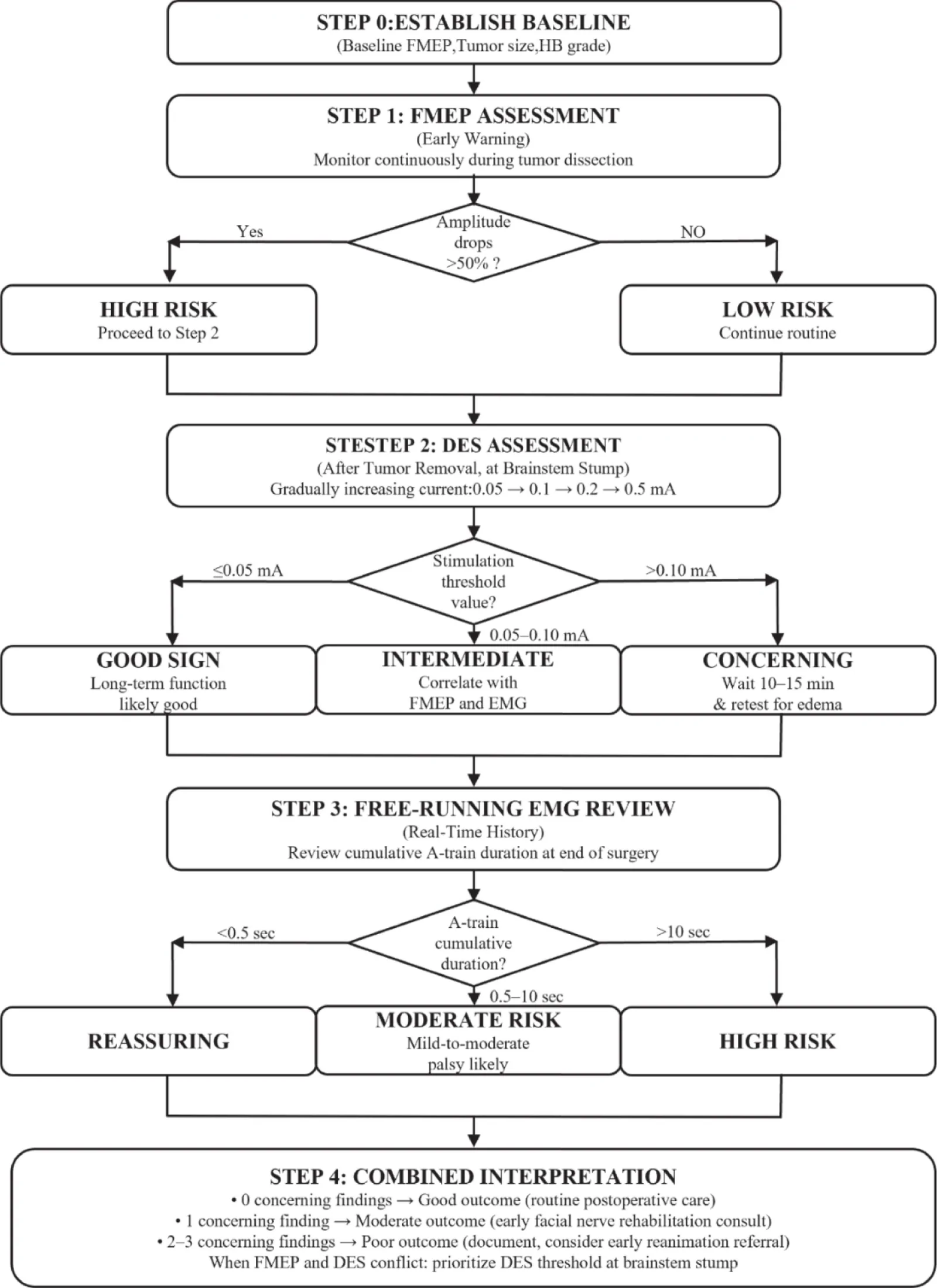
Stepwise Interpretation Framework for Intraoperative Facial Nerve Outcome Prediction. **Legend.** Stepwise interpretation framework for intraoperative facial nerve outcome prediction. Step 0: Baseline FMEP after dural opening. Step 1: FMEP monitoring during dissection. Step 2: DES assessment after tumor removal. Step 3: Free-running EMG review. Step 4: Combined interpretation. FMEP, facial motor evoked potential; DES, direct electrical stimulation; EMG, electromyography. Threshold values: FMEP amplitude reduction >50% (poor); DES threshold ≤0.05 mA (good) vs >0.10 mA (poor); A-train cumulative duration <0.5 s (reassuring) vs >10 s (high risk).

### 2.4 Outcome measures

The proposed framework predicts both short-term (within 2 weeks postoperatively) and long-term (≥6 months) facial nerve function, following the timepoint definitions used in prior meta-analyses (Quimby et al., 2021; Pan et al., 2024), with particular reference to the 1-year outcome (Patel et al., 2026).

## 3 Results

### 3.1 Facial Motor Evoked Potentials (FMEPs)

#### 3.1.1 Technical principle*.*

FMEPs are recorded by transcranial electrical stimulation of the motor cortex. The signal travels via the corticobulbar tract to activate the facial nerve and its innervated muscles, generating a compound muscle action potential (CMAP) (Acioly et al., 2010). Since their first report in skull base surgery in 2005 (Akagami et al., 2005), FMEPs have become standard in large acoustic neuroma surgery. FMEPs assess facial nerve functional integrity before the nerve is fully exposed during tumor dissection, providing earlier warning than DES (Della Pepa et al., 2022). A randomized controlled trial demonstrated that total intravenous anesthesia (TIVA) allows earlier recovery of neuromuscular function suitable for FMEP monitoring compared with sevoflurane anesthesia (Bang et al., 2025).

#### 3.1.2 Stimulation and recording parameters

FMEPs are elicited via transcranial electrical stimulation using corkscrew electrodes placed according to the international 10–20 system at C3/C4–Cz, C1/C2, or Cz-1/Cz + 6 (Szelényi et al., 2007); the FCC5h/FCC6h–Mz montage reduces peripheral facial nerve activation and improves specificity (Izzo et al., 2024). Stimuli consist of 3–5 rectangular pulses (0.05–0.5 ms duration, 2–4 ms interstimulus interval, 80–400 V) (Szelényi et al., 2007; Izzo et al., 2024). Bipolar subdermal needle electrodes record from orbicularis oculi, orbicularis oris, and optionally mentalis (Kartush et al., 2021).

Deletis described a “double-train” technique to distinguish central corticobulbar responses from peripheral facial nerve activation (Deletis et al., 2009; Deletis and Fernández-Conejero, 2016). A short train of 3–5 pulses is followed by a single pulse 90 ms later. Under general anesthesia, single pulses readily excite cranial nerves, whereas bulbar motor neurons require temporal summation. A response to the train alone confirms central origin; a response to the single pulse indicates peripheral spread and direct nerve stimulation—a confounder for corticobulbar monitoring. The double-train method provides an internal validity check. Our recommended FCC5h/FCC6h–Mz montage with short-train parameters achieves similar specificity via optimized electrode placement, without dual-train validation, and is simpler for routine use. Both techniques are complementary, sharing the same goal of minimizing peripheral current spread.

#### 3.1.3 Predictive parameter and threshold

The end-of-surgery FMEP amplitude ratio (final amplitude divided by maximum baseline amplitude) is the most reliable predictor of long-term facial nerve function. In a study of 106 patients with large acoustic neuromas, the FMEP ratio correlated highly with both immediate and long-term function (*P* < 0.001) (Liu et al., 2015). An FMEP ratio ≥ 60% predicts excellent 1-year function (House-Brackmann (HB) grade I-II) (Bhimrao et al., 2016).

A 2024 systematic review (17 studies, 1206 patients) reported that for predicting good long-term function (≥1 year), an end-of-surgery FMEP amplitude reduction of <50% had pooled sensitivity of 0.82 (95% CI, 0.74–0.88) and specificity of 0.93 (95% CI, 0.87–0.96) (Pan et al., 2024). A separate meta-analysis reached consistent conclusions (Zhang et al., 2024). Quantitative analysis of FMEP waveform parameters has refined predictive accuracy (Acioly et al., 2011).

#### 3.1.4 Clinical application

An amplitude reduction >50% at end of surgery indicates high risk for severe facial palsy (93% specificity). The moderate sensitivity (∼80%) means that preserved amplitude does not guarantee normal function. The 2026 CNS guidelines note that although FMEPs and free-running EMG provide continuous noninvasive monitoring, insufficient data exist to determine which correlates more strongly with facial function outcomes (Patel et al., 2026).

#### 3.1.5 Technical limitations

Residual neuromuscular blockade suppresses neuromuscular junction transmission, causing artificially reduced amplitudes (Lyon et al., 2005). FMEP monitoring requires TIVA with stable train-of-four count (Sala et al., 2007). Major warning criteria for facial nerve monitoring include an amplitude reduction >50% (MacDonald et al., 2013). Bang et al. (2025) showed that the median recovery index (time from end of neuromuscular blockade to train-of-four ratio ≥ 0.9) was 9 min with TIVA versus 34 min with sevoflurane (*P* < 0.001). FMEPs reflect entire motor pathway integrity. When injury is confined to the distal facial nerve (for example, within the internal auditory canal) while the proximal pathway remains intact, FMEPs may not sensitively reflect focal injury (Dong et al., 2005). Unlike free-running EMG, FMEP is sampled intermittently, as continuous stimulation may cause movement and disrupt surgery; thus it provides periodic rather than continuous assessment (Dong et al., 2005)**.** Stimulation parameters, recording electrode positions, and amplitude calculation methods vary across centers (Zachem et al., 2025).

### 3.2 Direct electrical stimulation (DES)

#### 3.2.1 Technical principle and parameters

DES uses a handheld probe to stimulate the nerve directly and elicit a CMAP. It remains the standard method for intraoperative nerve localization and verifying anatomical continuity (Dahm et al., 2025). Unlike FMEPs, DES provides focal information about the nerve segment distal to the stimulation point.

Two parameters guide prediction. The minimum stimulation threshold: the lowest current that elicits a recognizable CMAP reflects distal nerve excitability. The maximum CMAP amplitude with supramaximal stimulation reflects recruitable motor unit count and axonal integrity (Kimura, 2013).

#### 3.2.2 Stimulation and recording parameters

DES is performed using a handheld monopolar or bipolar probe with constant current stimulation and a return electrode on the ipsilateral shoulder or sternum. Stimuli are 0.05–0.1 ms rectangular pulses at 4 Hz; current is titrated from 0.05 mA upward (Kartush et al., 2021; Gazia et al., 2023). Bipolar subdermal needles record from orbicularis oculi and orbicularis oris (Kartush et al., 2021). The proximal brainstem stump is the key assessment site; testing should be performed after tumor removal (Gazia et al., 2023; Quimby et al., 2021).

#### 3.2.3 Predictive evidence and threshold

Three DES parameters predict good short-term function: (1) proximal (brainstem) stimulation threshold ≤0.04 mA, (2) proximal supramaximal CMAP amplitude >800 μV, and (3) proximal-to-distal CMAP amplitude ratio > 0.6 (Bernat et al., 2010). Subsequent studies refined these thresholds (Huang et al., 2018; Lee et al., 2016; Gazia et al., 2023).

A 2021 meta-analysis (21 studies, 1968 patients) provides large-scale evidence (Quimby et al., 2021). Both 0.05 mA and 0.10 mA thresholds at the brainstem stump significantly predicted good long-term (≥1 year) facial nerve function. A threshold ≤0.05 mA yielded sensitivity of 0.73 (95% CI, 0.58–0.84) and specificity of 0.74 (95% CI, 0.59–0.85). A threshold ≤0.10 mA yielded sensitivity of 0.76 (95% CI, 0.53–0.90) and specificity of 0.68 (95% CI, 0.42–0.87). That meta-analysis also found that a postoperative supramaximal CMAP amplitude <500 μV predicts short-term (within 1 week) severe facial palsy with high sensitivity. A prospective cohort study of 103 large tumors confirmed that response to 0.05 mA stimulation predicted better facial nerve outcomes (Huang et al., 2018).

#### 3.2.4 Clinical application

A threshold ≤0.05 mA at the brainstem stump is a useful rule-in test for good recovery. A threshold >0.10 mA raises concern but may reflect transient nerve edema rather than permanent injury.

#### 3.2.5 Technical limitations

Stimulation site and timing matter. This region is most often involved with tumor adhesion and vulnerable blood supply. Low-threshold stimulation at this site indicates preserved anatomical continuity and physiological conduction (Sughrue et al., 2010). Neurapraxia and nerve edema are common pitfalls. Even with intact axonal continuity, local edema may impair conduction, causing elevated thresholds or reduced amplitudes. Waiting 10–15 min after dissection allows nerve edema to partly resolve before final DES assessment (Neff et al., 2005). Stimulus parameter standardization remains an issue, with variations in electrode type, stimulus duration, and frequency across studies (Selesnick, 1999).

### 3.3 Free-running electromyography (EMG)

#### 3.3.1 Technical principle and classification

Free-running EMG continuously monitors spontaneous facial muscle activity, providing real-time feedback about mechanical, thermal, or traction stimuli. Unlike FMEPs and DES (evoked responses), free-running EMG is passive; it reflects spontaneous nerve activity in response to irritation.

Free-running EMG activity has five categories: burst, spike, A-train, B-train, and C-train (Romstöck et al., 2000). A-train activity: high-frequency (60–250 Hz), regular, sine wave-like synchronized discharges is the pattern most associated with new postoperative facial palsy. It reflects synchronized spontaneous discharges of injured motor unit muscle fibers (Nakao et al., 2001). The Best Practices consensus document emphasizes that A-train activity correlates significantly with postoperative facial palsy, whereas bursts and other patterns do not (Kartush et al., 2021).

#### 3.3.2 Stimulation and recording parameters

Free-running EMG is recorded continuously using bipolar subdermal needle electrodes placed 5–10 mm apart in the orbicularis oculi and orbicularis oris muscles ipsilateral to the surgical side. A minimal filter bandpass of 10–1000 Hz is suggested, with sensitivity ranging from 20 to 400 μV/division; sweep speeds of 30–200 ms/division are recommended for artifact differentiation. Audio output with squelch is essential for real-time feedback (Kartush et al., 2021).

#### 3.3.3 Predictive value and threshold

Romstöck et al. (2000) first reported that A-train activity predicted immediate postoperative facial palsy (sensitivity 86%, specificity 80%), though without correlating its amount with palsy severity. Prell et al. (2007) introduced cumulative A-train duration and found that >10 s predicted ≥2-grade deterioration (sensitivity 66.7%, specificity 88.0%). However, subsequent work by the same group revealed a non-linear, inverse sigmoid relationship between train time and palsy severity, cautioning against rigid thresholds (Prell et al., 2014). Frigeni et al. (2020) confirmed that the presence of A-train activity independently predicted poor 1-year function (odds ratio 9.81–11.72), but did not quantify duration.

#### 3.3.4 Clinical application

A cumulative A-train duration >10 s should raise serious concern for severe immediate palsy, particularly in patients with pre-existing dysfunction. Conversely, train time < 0.5 s is reassuring, and absence of A-trains in patients without preoperative weakness is strongly associated with good function (Prell et al., 2007). Given the non-linear relationship with palsy severity, the >10-s threshold is best used as a warning sign rather than a rigid predictor.

#### 3.3.5 Technical limitations

Pattern recognition remains subjective, depending on the experience of the monitoring technologist and neurophysiologist (Acioly et al., 2013). Strong mechanical stimuli may trigger brief burst activity without necessarily causing postoperative dysfunction. Conversely, slow blunt injury (for example, chronic tumor compression) may not elicit obvious EMG activity- the “silent injury” phenomenon (Harper, 2004).

### 3.4 Summary of thresholds

Table 1 summarizes the three modalities, their key parameters, evidence based thresholds for good versus poor outcomes, primary advantages, and technical limitations. Readers may use Table 1 as a rapid intraoperative reference.

### 3.5 Stepwise interpretation framework

The framework integrates three monitoring modalities chronologically according to their intraoperative sequence. Fig. 1 provides a visual decision aid.

#### 3.5.1 **Step 0: establish baseline**

When: After dural opening, before tumor manipulation.

Procedure: Record stable FMEP amplitudes from orbicularis oris and orbicularis oculi. Baseline values are defined as the mean of the two highest reproducible amplitudes obtained from at least three consecutive suprathreshold stimulations. Confirm train-of-four ≥3/4 under TIVA. Document preoperative House–Brackmann grade and note tumor size on preoperative imaging.

Interpretation: Markedly reduced or absent baseline amplitude suggests pre-existing dysfunction. In patients with preoperative weakness, thresholds should be interpreted with particular caution, as baseline function is a major prognostic factor that may outweigh intraoperative signals.

#### 3.5.2 **Step 1: FMEP Assessment (Early Warning).**

When: Every 15–30 min during tumor dissection; final assessment at closure.

Interpretation: End-of-surgery amplitude reduction >50% from baseline→ Predicts poor long-term function.

Action: Persistent reduction that does not recover with surgical pause and irrigation should prompt reassessment. A 10–15 min observation period helps distinguish reversible from irreversible changes. Preserved amplitude does not guarantee normal function.

#### 3.5.3 **Step 2: DES Assessment (Focal Integrity)**

When: After tumor resection, stimulate the proximal brainstem stump using gradually increasing current (0.05 mA → 0.1 mA → 0.2 mA → 0.5 mA).

Interpretation:

Thresholds≤0.05 mA → Predicts good long-term function.

Thresholds 0.05–0.10 mA → Intermediate; correlate with other modalities.

Thresholds >0.10 mA → Concerning; retest after 10–15 min as edema may resolve.

Action: A threshold >0.10 mA should prompt reassessment of resection extent versus nerve preservation. In densely adherent tumors, weigh electrophysiologic findings against observed anatomical adherence.

#### 3.5.4 **Step 3: Free-running EMG Review (Real-time History)**

When: Continuously throughout surgery; review cumulative A-train duration at closure.

Interpretation:

A-train duration <0.5 s → Reassuring.

A-train duration 0.5–10 s → Moderate concern.

A-train duration >10 s → High risk for severe immediate palsy.

Action: A-train >10 s should prompt heightened vigilance and documentation. Due to the non-linear relationship between train time and palsy severity, this threshold is a warning sign rather than a rigid predictor.

#### 3.5.5 **Step 4: Combined Interpretation**

A finding is defined as **concerning** if any of the following is present:

• FMEP: end-of-surgery amplitude reduction >50%
• DES: brainstem stump threshold >0.10 mA
• Free-running EMG: cumulative A-train duration >10 s

0 concerning findings →Good outcome. Recommend routine postoperative care.

1concerning finding →Moderate outcome (possible mild-to-moderate palsy). Recommend early facial nerve rehabilitation consult.

2 or 3 concerning findings → Poor outcome (high risk of severe palsy).

#### 3.5.6 When findings conflict

FMEP and DES sometimes disagree. Clinical context should guide interpretation; neither modality has been validated as superior in resolving discrepancies. In patients with dense tumor-nerve adhesion, both may underestimate traction injury risk; surgeons should weigh electrophysiologic data against observed adherence when deciding resection extent.

#### 3.5.7 Validation status

The 0/1/2–3 classification is based on the additive prognostic value of independent modalities. This specific classification has not been prospectively validated; thresholds are derived from the individual evidence base of each modality. Prospective studies are needed to test this combined framework.

#### 3.5.8 Clinical Caveats

**FMEPs are more reliable when:** Train-of-four count is stable (≥3/4), TIVA is used without volatile anesthetics (Bang et al., 2025), recordings are consistent, and amplitude drop is progressive.

**FMEPs are less reliable when:** Neuromuscular blockers were used, the patient has pre-existing facial weakness, or stimulation parameters changed.

**DES is more reliable when:** The proximal brainstem stump is tested after a 10–15 min waiting period, and thresholds are titrated from low to high (Kartush et al., 2021; Neff et al., 2005).

**DES is less reliable when:** Testing was performed immediately after aggressive manipulation, significant tissue edema is present, or the nerve is not clearly visible. In densely adherent tumors, preserved conduction does not exclude traction injury.

**Free-running EMG is more reliable when:** Classic A-train morphology is present, A-train persists for >10 s, and activity coincides with a specific surgical maneuver.

**Free-running EMG is less reliable when:** Only brief bursts or spikes (<0.5 s) are present, the pattern is ambiguous, or significant baseline noise is present.

## 4 Limitations and future directions

Several limitations of the synthesized evidence and the proposed framework merit explicit discussion.

**1. Evidence quality and heterogeneity.** The thresholds reported in this synthesis are derived from studies that are predominantly single-center and retrospective in design. Methodological heterogeneity across studies affects parameter definitions, stimulation techniques, and measurement protocols (Zachem et al., 2025). The 2026 CNS guidelines note that all available IONM studies are graded as Class III evidence (Patel et al., 2026). Publication bias may overestimate predictive values. Despite these limitations, the synthesized thresholds represent the best available evidence for intraoperative interpretation.

**2. Generalizability to patients with preoperative facial weakness.** The primary threshold values (FMEP >50% reduction, DES ≤0.05 mA, A-train >10 s) are derived from studies that predominantly excluded patients with preoperative HB III-VI (Quimby et al., 2021; Pan et al., 2024). For patients with pre-existing weakness, baseline function is a major prognostic factor that may outweigh intraoperative signals; the proposed thresholds should therefore be interpreted with particular caution in this subgroup. Moreover, the prognostic value of anatomically intact but electrophysiologically silent nerves has not been systematically evaluated; this scenario is not uncommon in large or recurrent tumors.

**3. Anatomical factors not incorporated.** Tumor size and nerve-tumor adhesion are established predictors of postoperative facial palsy (Xia et al., 2025; Tawfik et al., 2020). The current framework does not incorporate these preoperative or intraoperative anatomical factors. Surgeons should integrate electrophysiological findings with tumor characteristics and adhesion severity when considering extent of resection, particularly in large or cystic tumors where near-total resection may balance function preservation against recurrence risk (Taha et al., 2020).

**4. Unvalidated combined framework.** The proposed 0/1/2–3 classification (Fig. 1) has not been prospectively validated. The assignment of outcome categories is based on the independent predictive performance of each modality, not on formal diagnostic accuracy data for the combination. The A-train threshold of >10 s derives from a single-center study with a modest sample size (Prell et al., 2007), and subsequent work by the same group described a non-linear, inverse sigmoid relationship between train time and palsy severity, cautioning against rigid thresholds (Prell et al., 2014). We therefore present this threshold as a warning sign rather than a definitive predictor. Prospective multicenter studies are required to test the framework's predictive utility.

**5. Omitted parameters.** DES latency measures were not incorporated into the framework. Although latency may provide additional information about conduction block, no meta-analysis or large-scale study has reported latency-based thresholds for intraoperative prediction (Quimby et al., 2021; no latency data reported). Similarly, the blink reflex, recently reported as an independent predictor of postoperative facial dysfunction (Abdelgadir et al., 2026) was not included as a core modality due to limited comparative data with the three established techniques.

**6. Time interval between diagnosis and surgery.** This interval was not incorporated. Its influence is largely mediated by tumor size and baseline facial nerve function, both included in the framework, and its independent contribution has not been established in the source literature.

**Future directions:** Three areas merit further investigation. First, standardized protocols from multicenter prospective studies would reduce heterogeneity (Patel et al., 2026; Kartush et al., 2021). Second, dynamic prediction models incorporating preoperative, intraoperative, and postoperative data may improve accuracy. Third, automated A-train identification using artificial intelligence is an emerging area (Przepiorka et al., 2025; Hajikarimloo et al., 2025), and the blink reflex warrants further evaluation as an adjunctive modality.

## 5 Conclusions

This report provides a stepwise framework that integrates FMEP amplitude ratio, DES threshold at the brainstem stump, and cumulative A-train duration to guide intraoperative prediction of facial nerve function after acoustic neuroma surgery. An FMEP reduction >50% at closure warns of poor long-term function with high specificity; a DES threshold ≤0.05 mA reliably predicts good recovery; and an A-train duration exceeding 10 s signals elevated risk of severe immediate palsy, whereas <0.5 s is reassuring (Prell et al., 2007). No single parameter suffices; the combined interpretation based on the number of concerning findings across the three modalities which provides a more balanced assessment. The framework emphasizes baseline function as a key prognostic anchor, particularly in patients with preoperative weakness, and acknowledges that anatomical factors such as tumor size and nerve adhesion must be weighed alongside electrophysiological data when considering resection extent. The 2026 CNS guidelines support routine IONM use and confirm that electrophysiological parameters predict long-term outcomes better than clinical information alone (Patel et al., 2026). Table 1 and Fig. 1 offer a visual decision aid and rapid intraoperative reference. Prospective multicenter validation of this combined framework is needed.

## Declaration of competing interest

The authors declare that they have no known competing financial interests or personal relationships that could have appeared to influence the work reported in this paper.

## References

[bb0005] Abdelgadir D., Karsy M., Couldwell M. (2026). The role of the blink reflex in predicting facial nerve outcomes following vestibular schwannoma surgery: a 4-year retrospective review. J. Neurosurg..

[bb0010] Acioly M.A., Liebsch M., Carvalho C.H., Gharabaghi A., Tatagiba M. (2010). Transcranial electrocortical stimulation to monitor the facial nerve motor function during cerebellopontine angle surgery. Neurosurgery.

[bb0015] Acioly M.A., Gharabaghi A., Liebsch M. (2011). Quantitative parameters of facial motor evoked potential during vestibular schwannoma surgery predict postoperative facial nerve function. Acta Neurochir..

[bb0020] Acioly M.A., Liebsch M., de Aguiar P.H., Tatagiba M. (2013). Facial nerve monitoring during cerebellopontine angle and skull base tumor surgery: a systematic review from description to current success on function prediction. World Neurosurg..

[bb0025] Akagami R., Dong C.C., Westerberg B.D. (2005). Localized transcranial electrical motor evoked potentials for monitoring cranial nerves in cranial base surgery. Neurosurgery.

[bb0030] Bang Y.J., Lee S.H., Jeong Y.W. (2025). Effect of total intravenous anesthesia versus sevoflurane anesthesia on intraoperative facial nerve monitoring: a randomized controlled trial. Can. J. Anesth..

[bb0035] Bernat I., Grayeli A.B., Esquia G., Zhang Z., Kalamarides M., Sterkers O. (2010). Intraoperative electromyography and surgical observations as predictive factors of facial nerve outcome in vestibular schwannoma surgery. Otol. Neurotol..

[bb0040] Bhimrao S.K., Le T.N., Dong C.C. (2016). Role of facial nerve motor-evoked potential ratio in predicting facial nerve function in vestibular schwannoma surgery both immediate and at 1 year. Otol. Neurotol..

[bb0045] Carlson M.L., Link M.J. (2021). Vestibular schwannomas. N. Engl. J. Med..

[bb0050] Deletis V., Fernández-Conejero I. (2016). Intraoperative monitoring and mapping of the functional integrity of the brainstem. J. Clin. Neurol..

[bb0055] Deletis V., Fernandez-Conejero I., Ulkatan S., Costantino P. (2009). Methodology for intraoperatively eliciting motor evoked potentials in the vocal muscles by electrical stimulation of the corticobulbar tract. Clin. Neurophysiol..

[bb0060] Delgado T.E., Bucheit W.A., Rosenholtz H.R., Chrissian S. (1979). Intraoperative monitoring of facial muscle evoked responses obtained by intracranial stimulation of the facial nerve. Neurosurgery.

[bb0065] Della Pepa G.M., Stifano V., D’Alessandris Q.G. (2022). Intraoperative corticobulbar motor evoked potential in cerebellopontine angle surgery: a clinically meaningful tool to predict early and late facial nerve recovery. Neurosurgery.

[bb0070] Dong C.C., Macdonald D.B., Akagami R. (2005). Intraoperative facial motor evoked potential monitoring with transcranial electrical stimulation during skull base surgery. Clin. Neurophysiol..

[bb0075] Freeman S.R.M., Kannan R., Nduka C. (2025). Prevention and rehabilitation of facial palsy in patients with vestibular schwannomas. Handb. Clin. Neurol..

[bb0080] Frigeni B., Bivona R., Foresti C., Guazzo E., Danesi G. (2020). Predictive value of preoperative and intraoperative neurophysiology in evaluating long-term facial function outcome in acoustic neuroma surgery. Otol. Neurotol..

[bb0085] Gazia F., Callejo A., Pérez-Grau M. (2023). Pre-and intra-operative prognostic factors of facial nerve function in cerebellopontine angle surgery. Eur. Arch. Otorhinolaryngol..

[bb0090] Hajikarimloo B., Mohammadzadeh I., Nazari M.A. (2025). Prediction of facial nerve outcomes after surgery for vestibular schwannoma using machine learning-based models: a systematic review and meta-analysis. Neurosurg. Rev..

[bb0095] Harper C.M. (2004). Intraoperative cranial nerve monitoring. Muscle Nerve.

[bb0100] Huang X., Ren J., Xu J. (2018). The utility of “low current” stimulation threshold of intraoperative electromyography monitoring in predicting facial nerve function outcome after vestibular schwannoma surgery: a prospective cohort study of 103 large tumors. J. Neurooncol.

[bb0105] Izzo M.G.A., Rossi Sebastiano D., Catanzaro V. (2024). Three montages for transcranial electric stimulation in predicting the early post-surgery outcome of the facial nerve functioning. Clin. Neurophysiol..

[bb0110] Kartush J.M., Rice K.S., Minahan R.E., Balzer G.K., Yingling C.D., Seubert C.N. (2021). Best practices in facial nerve monitoring. Laryngoscope.

[bb0115] Kimura J. (2013).

[bb0120] Lee S., Seol H., Park K. (2016). Functional outcome of the facial nerve after surgery for vestibular schwannoma: prediction of acceptable long-term facial nerve function based on immediate postoperative facial palsy. World Neurosurg..

[bb0125] Liu S.W., Jiang W., Zhang H.Q. (2015). Intraoperative neuromonitoring for removal of large vestibular schwannoma: facial nerve outcome and predictive factors. Clin. Neurol. Neurosurg..

[bb0130] Lyon R., Feiner J., Lieberman J.A. (2005). Progressive suppression of motor evoked potentials during general anesthesia: the phenomenon of “anesthetic fade”. J. Neurosurg. Anesthesiol..

[bb0135] MacDonald D.B., Skinner S., Shils J., Yingling C. (2013). Intraoperative motor evoked potential monitoring — a position statement by the American Society of Neurophysiological Monitoring. Clin. Neurophysiol..

[bb0140] Nakao Y., Piccirillo E., Falcioni M., Taibah A., Kobayashi T., Sanna M. (2001). Electromyographic evaluation of facial nerve damage in acoustic neuroma surgery. Otol. Neurotol..

[bb0145] Neff B.A., Ting J., Dickinson S.L., Welling D.B. (2005). Facial nerve monitoring parameters as a predictor of postoperative facial nerve outcomes after vestibular schwannoma resection. Otol. Neurotol..

[bb0150] Pan S.Y., Holdefer R.N., Wu H.L., Li C.R., Guo L. (2024). The predictive value of intraoperative facial motor evoked potentials in cerebellopontine angle tumor surgery. Clin. Neurophysiol..

[bb0155] Patel N.S., Carlson M.L., Sughrue M.E., Olson J.J. (2026). Congress of Neurological Surgeons systematic review and evidence-based guidelines update for the role of intraoperative cranial nerve monitoring in the Management of Patients with Vestibular Schwannomas. Neurosurgery.

[bb0160] Prell J., Rampp S., Romstock J., Fahlbusch R., Strauss C. (2007). Train time as a quantitative electromyographic parameter for facial nerve function in patients undergoing surgery for vestibular schwannoma. J. Neurosurg..

[bb0165] Prell J., Strauss C., Rachinger J. (2014). Facial nerve palsy after vestibular schwannoma surgery: dynamic risk-stratification based on continuous EMG-monitoring. Clin. Neurophysiol..

[bb0170] Przepiorka L., Kujawski S., Wojtowicz K., Maj E., Marchel A., Kunert P. (2025). Development and application of explainable artificial intelligence using machine learning classification for long-term facial nerve function after vestibular schwannoma surgery. J. Neurooncol.

[bb0175] Quimby A.E., Lui J., Chen J. (2021). Predictive ability of direct electrical stimulation on facial nerve function following vestibular schwannoma surgery: a systematic review and meta-analysis. Otol. Neurotol..

[bb0180] Romstock J., Strauss C., Fahlbusch R. (2000). Continuous electromyography monitoring of motor cranial nerves during cerebellopontine angle surgery. J. Neurosurg..

[bb0185] Saadi R., Shokri T., Schaefer E. (2019). Depression rates after facial paralysis. Ann. Plast. Surg..

[bb0190] Sala F., Manganotti P., Tramontano V., Bricolo A., Gerosa M. (2007). Monitoring of motor pathways during brain stem surgery: what we have achieved and what we still miss?. Neurophysiol. Clin..

[bb0195] Selesnick S.H. (1999). Optimal stimulus duration for intraoperative facial nerve monitoring. Laryngoscope.

[bb0200] Sughrue M.E., Kaur R., Kane A.J. (2010). The value of intraoperative facial nerve electromyography in predicting facial nerve function after vestibular schwannoma surgery. J. Clin. Neurosci..

[bb0205] Szelényi A., Kothbauer K.F., Deletis V. (2007). Transcranial electric stimulation for intraoperative motor evoked potential monitoring: stimulation parameters and electrode montages. Clin. Neurophysiol..

[bb0210] Taha I., Hyvärinen A., Ranta A. (2020). Facial nerve function and hearing after microsurgical removal of sporadic vestibular schwannomas in a population-based cohort. Acta Neurochir..

[bb0215] Tawfik K.O., Alexander T.H., Saliba J., Mastrodimos B., Cueva R.A. (2020). Predicting long-term facial nerve outcomes after resection of vestibular schwannoma. Otol. Neurotol..

[bb0220] Xia D., Zhang Z., Shen J., Ge R., Tan M., Jin Y. (2025). Study on risk factors affecting facial nerve function after microsurgical resection of acoustic neuroma. J. Craniofac. Surg..

[bb0225] Zachem T.J., Bello A., Lee B. (2025). Current state of intraoperative neuromonitoring of the facial nerve during skull base surgery: a systematic review. J. Clin. Neurosci..

[bb0230] Zhang H.X., Zong Y.X., Hu Y. (2024). Predictive value of intraoperative facial nerve motor evoked potentials for facial nerve function in patients with cerebellopontine angle tumors: a meta-analysis. Zhonghua Shen Jing Wai Ke Za Zhi..

